# Imaging free radicals in organelles, cells, tissue, and in vivo with immuno-spin trapping

**DOI:** 10.1016/j.redox.2016.04.003

**Published:** 2016-04-22

**Authors:** Ronald Paul Mason

**Affiliations:** Immunity, Inflammation, and Disease Laboratory, National Institute of Environmental Health Sciences, NIH, Research Triangle Park, NC 27709, USA

**Keywords:** Immuno-spin trapping, Free radical detection, Spin trap, Mass spectrometry, Confocal microscopy, Molecular resonance imaging

## Abstract

The accurate and sensitive detection of biological free radicals in a reliable manner is required to define the mechanistic roles of such species in biochemistry, medicine and toxicology. Most of the techniques currently available are either not appropriate to detect free radicals in cells and tissues due to sensitivity limitations (electron spin resonance, ESR) or subject to artifacts that make the validity of the results questionable (fluorescent probe-based analysis). The development of the immuno-spin trapping technique overcomes all these difficulties. This technique is based on the reaction of amino acid- and DNA base-derived radicals with the spin trap 5, 5-dimethyl-1-pyrroline *N*-oxide (DMPO) to form protein- and DNA-DMPO nitroxide radical adducts, respectively. These adducts have limited stability and decay to produce the very stable macromolecule-DMPO-nitrone product. This stable product can be detected by mass spectrometry, NMR or immunochemistry by the use of anti-DMPO nitrone antibodies. The formation of macromolecule-DMPO-nitrone adducts is based on the selective reaction of free radical addition to the spin trap and is thus not subject to artifacts frequently encountered with other methods for free radical detection. The selectivity of spin trapping for free radicals in biological systems has been proven by ESR. Immuno-spin trapping is proving to be a potent, sensitive (a million times higher sensitivity than ESR), and easy (not quantum mechanical) method to detect low levels of macromolecule-derived radicals produced in vitro and in vivo. Anti-DMPO antibodies have been used to determine the distribution of free radicals in cells and tissues and even in living animals. In summary, the invention of the immuno-spin trapping technique has had a major impact on the ability to accurately and sensitively detect biological free radicals and, subsequently, on our understanding of the role of free radicals in biochemistry, medicine and toxicology.

## Highlights

• Immuno-spin trapping detects protein and DNA radicals in cells, tissue and in vivo.
• Free radicals from proteins can be distinguished from those from DNA without MS.
• With MS free radicals from tyr, cys, his, trp and adenine were identified.

## Graphical abstract

**Figure:**
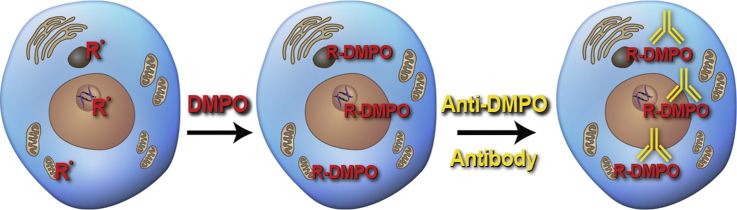

I was trained in the physical sciences as a physical chemist. Especially in physics, inductive reasoning is commonly used where general principles are derived from particular facts. In my biomedical research, the first question I ask is whether free radical formation is possible. This is really a question of thermodynamics, which can be calculated, but I rely on my chemical intuition. The next step is detecting the free radical. Before I invented immuno-spin trapping, I relied on ESR for this. Once the free radical is detected by ESR, the same experiment can, in general, identify the free radical. The next question is what are the reactions and the rate constants of these free radicals with oxygen, antioxidants, biochemicals, and macromolecules. A vast array of these rate constants have been determined by pulse radiolysis and other techniques. The last question is the most difficult question. What are the critical biological targets of the free radicals? In practice, I work backwards using deductive logic from the biochemical, toxicological and pathological consequences to discern the critical target and, ultimately, the initiating free radical event.

Many of the best understood human toxicities are generally accepted to be caused by free radicals. These toxicities include ionizing radiation, iron sulfate (the leading cause of pediatric poisoning), oxygen toxicity (common in premature infants), paraquat (classic pulmonary toxicant), daunorubicin (a cardiotoxicant), UVA radiation (skin cancer), and carbon tetrachloride (classic hepatotoxicant). Of these established free radical toxicities in humans, ESR experiments provided evidence that free radical formation is the fundamental, initiating event in all of them. On the other hand, the role of free radicals in human diseases is less definitive, largely because ESR has been unsuccessful in detection of free radicals in disease models. Presumably, in animal models of human diseases, free radical formation is characterized by lower rates of formation over a longer period of time than in acute toxicity models, and a technique much more sensitive than ESR was necessary to demonstrate free radical formation.

The advantages of free radical detection with ESR, which is without question the gold standard of free radical detection, are listed in Table 1. The disadvantages of ESR are listed in Table 2. In the biomedical sciences, the greatest limitation is the quantum mechanical basis of ESR. Quantum mechanics requires higher math and physics. People not trained in ESR have, in fact, been limited to repeating variations of experiments first done by people trained as ESR spectroscopists or, more commonly, totally excluded from the field. With the help of co-workers and collaborators, I have invented a technique that solves all these problems!

It starts with ESR and spin trapping.



Spin trapping is a technique in which a short-lived reactive free radical combines with a diamagnetic molecule (“spin trap”) to form a more stable free radical (“radical adduct”) which, historically, could only be detected by electron spin resonance (ESR). By extending the lifetime of the radical adduct, the concentration of the radical adduct is increased and, therefore, the signal-to-noise of the ESR spectrum. To an ESR spectroscopist, the conservation of the unpaired electron is the most important aspect of this reaction. To an organic chemist, the most unique feature of the reaction is the formation of a new chemical bond to the free radical in a way that is specific to free radicals.

Historically, my laboratory has used ESR, especially spin trapping, to detect free radicals. The most versatile and therefore most popular spin trap is DMPO (5,5-dimethyl-1-pyrroline *N*-oxide). The ESR spectrum of Mb^•^-DMPO is consistent with that of a partially immobilized nitroxide (Fig. 1A). Interpreting this ESR spectrum demonstrated that metmyoglobin reacts with hydrogen peroxide to produce a tyrosyl radical which is trapped by DMPO at the phenoxyl oxygen as demonstrated by O-17 isotope labeling [1], [2]. The trapping of this myoglobin radical is the result of hydrogen peroxide-driven self-peroxidation, which forms a phenoxyl radical at tyrosine-103 as determined by ESR studies using site-specific mutants where phenylalanine was substituted for tyrosine [2]. This radical adduct decays with a half-life of one minute [3].

After the ESR signal disappeared, analysis of the Mb-DMPO samples by electrospray ionization mass spectroscopy (MS) demonstrated the formation of a myoglobin-derived product with a mass increase of 111 Da, which is essentially the mass of DMPO (Fig. 1B). These data are consistent with the addition of DMPO as expected for the formation of a covalent bond between myoglobin and DMPO. Approximately one quarter of the myoglobin reacted to form the persistent DMPO adduct. This ion was not detected in the controls. This result always fascinated me, because this mass spectrum demonstrated that the ESR-silent species was still a chemical adduct of DMPO and myoglobin. These findings show that the DMPO remains covalently bound to the myoglobin (Mb-DMPO) after the ESR signal of the radical adduct decays. The oxidation of the radical adduct to a nitrone adduct is facile and expected on chemical grounds due to the ease of removal of the β-hydrogen to form the chemically stable nitrone adduct. The position of the DMPO nitrone adduct is a *specific marker or tag for where the radical was*, as determined by the new chemical bond formed during spin trapping.



I had the idea of making antibodies to the DMPO bound to proteins. In order to raise antiserum that specifically binds to a protein-DMPO adduct, it was necessary to synthesize a DMPO-nitrone protein conjugate. This required the synthesis of a DMPO-nitrone hapten, then linking it to a carrier protein [1]. The nitrone group is unknown in nature and should be highly antigenic, as is the related nitro group. So now with the anti-DMPO antibody, immunology instead of physics can be used to rigorously detect free radicals. Western blot staining revealed that this serum, diluted 1:5000, tested positive for Mb-DMPO (Fig. 1C). No detectable antibodies were bound to the control samples. Anti-DMPO antibodies bind to DMPO adducts with high affinity and, for a chemist, unbelievable specificity. The anti-DMPO antibody recognizes DMPO alone. Up to 50 mM DMPO is usually nontoxic [4], [5], and distributes to the heart, liver [6] and even the brain [7].

One of our early examples was Hb oxidation to radicals [8]. The reaction between metHb and H_2_O_2_ produces Hb-derived radicals that, in the presence of DMPO, form radical adducts ESR detectable at 0.375 mM Hb and 1.5 mM hydrogen peroxide concentrations [9], whereas with anti-DMPO. Hb-derived radical could be detected at 2 µM hydrogen peroxide and 1 µM metHb. Since radical formation is bimolecular with metHb and hydrogen peroxide, the flux of radical formation is about a million-fold lower with anti-DMPO detection. Hb-derived radical was also detected within red blood cells at 50 µM hydrogen peroxide in spite of the high concentrations of catalase and glutathione peroxidase present [8]. To determine the site of free radical formation in oxidized Hb, we analyzed the reaction mixtures by electrospray ionization MS as part of an ongoing collaboration with Leesa Deterding (Fig. 2). The electrospray ionization mass spectrum of the oxidized Hb shows one adduct each on both the alpha chain and the beta chain of hemoglobin, corresponding in mass to the addition of one DMPO molecule [10]. The identity of the radicals formed on hemoglobin was determined using proteolysis techniques followed by LC/MS and MS/MS analyses. Four sites of DMPO addition were identified on hemoglobin: Cys-93 of the beta chain, and Tyr-42, Tyr-24, and His-20 of the alpha chain (Fig. 2). The His-20 radical is the first discovered in a hemoprotein. Quantitative MS found only modest free radical redistribution among the amino acids within the oxidized hemoglobin when complexed with haptoglobin, and no DMPO adducts on the haptoglobin, which was unexpected [11].

The first localization of free radicals within an organelle was detected as the HOCl-induced, catalase free radical within mouse hepatocytes done with anti-DMPO confocal imaging (Fig. 3). Catalase is a major peroxisomal protein, and the co-localization of the anti-DMPO and the anti-catalase antibodies as punctuated spots is clear. This pattern is consistent with the fact that catalase is confined to the peroxisomes and that HOCl diffused through these organelles to oxidize catalase. In the absence of DMPO (Fig. 3C) or HOCl (Fig. 3D), catalase was still easily detectable, but no green fluorescein staining was observed due to the absence of anti-DMPO antibody binding. These data indicate that protein-radical formation was a consequence of the HOCl-induced catalase oxidation. Comparison with cells obtained from knockout mice gave no anti-DMPO staining in the cytosol (Fig. 3E and F). Thus, for the first time protein radical formation was localized to an organelle, the peroxisome, in a single cell. To confirm catalase as an important target of HOCl in cells, catalase was immunoprecipitated from mouse hepatocytes exposed to different HOCl concentrations before lysis. The protein that was recovered from the immunoprecipitates and the catalase radical was detected through Western blot with anti-DMPO antibody [12].

In our most recent work with cells [13], Fig. 4, MCF-7 breast cancer cells overexpressing SOD2 which were cultivated in regular media showed higher protein radical generation in the mitochondria when exposed to paraquat (an intracellular generator of superoxide and hydrogen peroxide). Under these conditions, SOD2 incorporated iron, acquiring peroxidase activity, a gain of function that enables SOD2 to utilize H_2_O_2_ to oxidize other molecules including itself. Cells with FeSOD2 show a shift in metabolism from oxidative phosphorylation to glycolysis due to mitochondrial dysfunction, and higher susceptibility to oxidative stress [13].

The first use of anti-DMPO to image free radicals using tissue immunohistochemistry was in the motor neurons of an animal model for amyotrophic lateral sclerosis [14], Fig. 5. Earlier reports indicated that astrocytes expressing the mutations of superoxide dismutase-1 (SOD1) contribute to motor neuron degeneration in amyotrophic lateral sclerosis. Spinal cord sections revealed an increase in DMPO-protein adduct staining in motor neurons and microglia cells from transgenic animals [14].

Alpha-synuclein-containing aggregates represent a feature of a variety of neurodegenerative disorders, including Parkinson's disease. However, the mechanism that initiates and promotes intraneuronal alpha-synuclein aggregation remains unknown. We hypothesized protein radicals as an initiating mechanism for alpha-synuclein aggregation [7]. Therefore, we used anti-DMPO to investigate protein radical formation as a possible mechanism of alpha-synuclein aggregation as well as to investigate the source of protein radical formation in the midbrains of Maneb (manganese ethylene-1,2-bisdithiocarbamate)- and paraquat-coexposed mice, a model of Parkinson's disease. Coexposure to Maneb and paraquat for 6 weeks resulted in microglia action, NADPH oxidase activation, and inducible nitric oxide synthase induction, which culminated in peroxynitrate-mediated protein radical formation in the midbrains of mice (Fig. 6). Results obtained with immuno-spin trapping and immunoprecipitation experiments confirmed formation of alpha-synuclein radicals in dopaminergic neurons of exposed mice. This free radical formation requires NADPH oxidase and inducible nitric oxide synthase [7]. Concurrence of protein radical formation with dopaminergic neuronal death indicated a link between protein radicals and disease progression. Taken together, these results show for the first time the formation and detection of the alpha-synuclein radical and suggested that NADPH oxidase and inducible nitric oxide synthase play roles in peroxynitrite-mediated protein radical formation and subsequent neuronal death in the midbrains of Maneb- and paraquat-coexposed mice [7].

Ever since the invention of MRI, the in vivo imaging of reactive free radicals has been a goal of ESR spectroscopists. Humans are mainly water, which is over 100 M in protons, whereas in vivo free radicals exist in a steady-state concentration less than nanomolar and probably much less. This over eleven orders of magnitude difference in the concentration of these spins has made the ESR detection of even very stable radical adducts, let alone the imaging, very challenging and has resulted in limited applications. In a paradigm shift, Rheal Towner used Molecular MRI (mMRI), which relies on the specific labeling of extracellular cell-surface protein antigens with a magnetic resonance contrast agent containing the anti-DMPO antibody, to obtain this long sought goal [15]. The anti-DMPO antibody-targeting probe alters proton magnetic relaxation times at their sites of accumulation. The contrast agent, the paramagnetic gadolinium (Gd), generates a positive signal contrast (T1 contrast), which enhances magnetic resonance signal intensities of water molecules that surround the Gd-based contrast agents in T1-weighted magnetic resonance images. This approach has been successful with a number of disease models including diabetes [15], amyotrophic lateral sclerosis [16], grafted GL261 gliomas [17], and septic encephalopathy [18], Fig. 7. Even quantitation has been done in vivo, with anti-DMPO probe concentrations being calculated from T1 relaxation differences in amyotrophic lateral sclerosis mice in the lumbar regions of mouse spinal cords. Estimated anti-DMPO probe concentrations were 103 µM in amyotrophic lateral sclerosis mice, which was significantly increased over controls [16].

Dario Ramirez knew that DNA could be made to stick to ELISA plates and that, therefore, radical detection could be extended to DNA because DNA radicals, like protein radicals, react with DMPO forming covalently-linked radical adducts [19]. The oxidized form of these adducts, nitrone adducts, are stable during standard DNA extraction procedures and are detected using heterogeneous immunoassays with the anti-DMPO antibody. The fact that DNA can easily be purified to very high homogeneity means that DNA radicals can easily be distinguished from the protein radicals. The strategy for detection of DNA radicals is basically the same. Initially we studied the reaction of hydroxyl radical with DNA. This reaction is very complicated, forming over one hundred products of DNA oxidation, the most popular of which is 8-oxo-dG, which we have measured immunologically to compare it to anti-DMPO [19]. A copper-driven Fenton system was used to produce hydroxyl radicals which formed 8-oxo-dG whereas, in the presence of DMPO, DNA radicals formed DNA nitrone adducts. Both assays were dependent on hydrogen peroxide concentration. Under these conditions, we observed that the anti-DMPO was sensitive at 50-fold lower concentrations of hydrogen peroxide then the 8-oxo-dG antibody (Fig. 8). Nuclear staining by anti-DMPO in the nucleus of cells treated with Cu(II)/H_2_O_2_ was first distinguished from protein radicals by DNA purification [19] and imaging [20], Fig. 9. Adenine base radical formation was proven using MS/MS [21]. Other DNA adducts were presumably formed but not detected by MS, which is much less sensitive than ELISA or confocal microscopy. Originally DNA radicals were detected only by ELISA, dot-blot assay, or confocal microscopy because the strongly alkaline conditions traditionally used in transferring DNA to a nitrocellulose membrane breaks the bond between DMPO and DNA bases. Therefore, we have developed immunoblotting methods for detection of DNA nitrone adducts on electrophoretically separated DNA, comparable to Western blotting for proteins [22]. These new techniques not only allow the assessment of relative radical adduct levels but can reveal specific DNA fragments and, ultimately, nucleotides as radical targets. Moreover, it was found that denaturation of samples into single stranded DNA enhances the detection of DNA-DMPO adducts in our new blotting methods and also in ELISA [22].

In summary, immuno-spin trapping combines the selectivity of spin trapping with the sensitivity of immunological techniques. For the first time, free radical formation can be localized in sub-cellular compartments and even in vivo. Unlike ESR, detection of free radicals is not dependent on transient free radical intermediates and is as much as a million times more sensitive. With the exception of peroxyl radicals [23], all classes of macromolecular free radicals known to exist in biological systems form stable DMPO nitrone adducts. Rigorous free radical detection is no longer dependent on an understanding of quantum mechanics and is now available to all biomedical investigators. A major limitation of the anti-DMPO antibody is the antibody target is DMPO and not a particular protein or DNA sequence, so the chemical nature of the free radical is unknown. At present, the investigator needs to characterize the chemical structure of the free radical by mass spectrometry, ESR, NMR or other molecular techniques. Therefore, an important advance would be the development of a free radical specific antibody, which could, for instance, distinguish DMPO bound to cysteine from DMPO bound to other biochemicals. A general challenge in free radical chemistry is to distinguish the initial free radical from secondary and even tertiary free radical formation. With immuno-spin trapping, this has been done by examining the effect of DMPO concentration where at the highest DMPO concentration, >100 mM, all of the primary free radical will be trapped, whereas at lower DMPO concentrations some primary free radical can react to form secondary free radicals which are, in their turn, trapped [24], [25]. The chemical and biological rationale for the anti-DMPO assay has been reviewed in detail [26], as have protocols [27], [28] and various experiments [29], [30].

## Figures and Tables

**Fig. 1:**
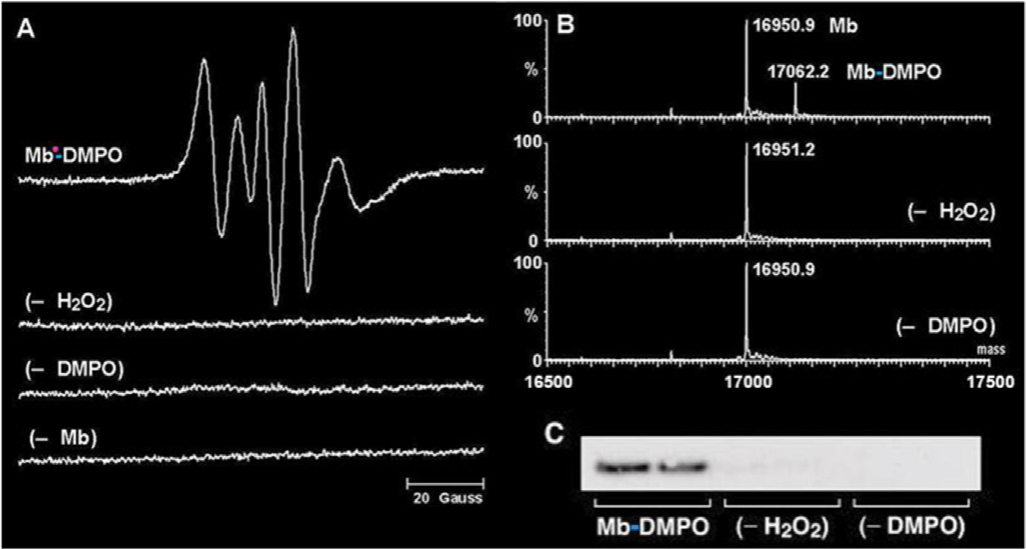
DMPO spin trapping of the tyrosyl radical generated on horse metmyoglobin by hydrogen peroxide. Samples were analyzed using ESR, ESI/MS, and Western blot, respectively. (A) ESR analysis: Sample contained 50 µM metmyoglobin, 50 µM hydrogen peroxide, and 10 mM DMPO. (B) ESI/MS analysis: Sample contained 1 µM metmyoglobin, 1 µM hydrogen peroxide, and 10 mM DMPO. (C) Western blot: Sample contained 5 µM metmyoglobin, 5 µM hydrogen peroxide, and 10 mM DMPO (1).

**Fig. 2:**
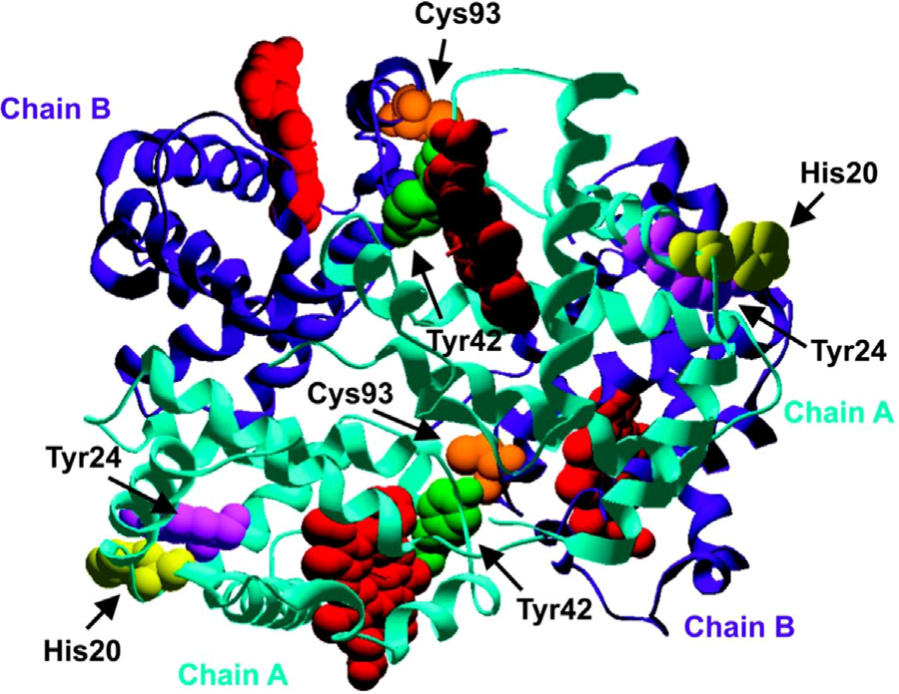
Amino acid residues trapped by DMPO as determined by mass spectrometry. The figure was generated from the crystal structure of the hemoglobin tetramer. The alpha chains are shown in *aqua*, the beta chains are shown in *purple*, and the hemes are shown in *red*. The Cys-93 of the beta chains is shown in *orange*, and the His-20, Tyr-24, and Tyr-42 of the alpha chains are shown in *yellow*, *violet*, and *green*, respectively (10). (For interpretation of the references to color in this figure legend, the reader is referred to the web version of this article.)

**Fig. 3:**
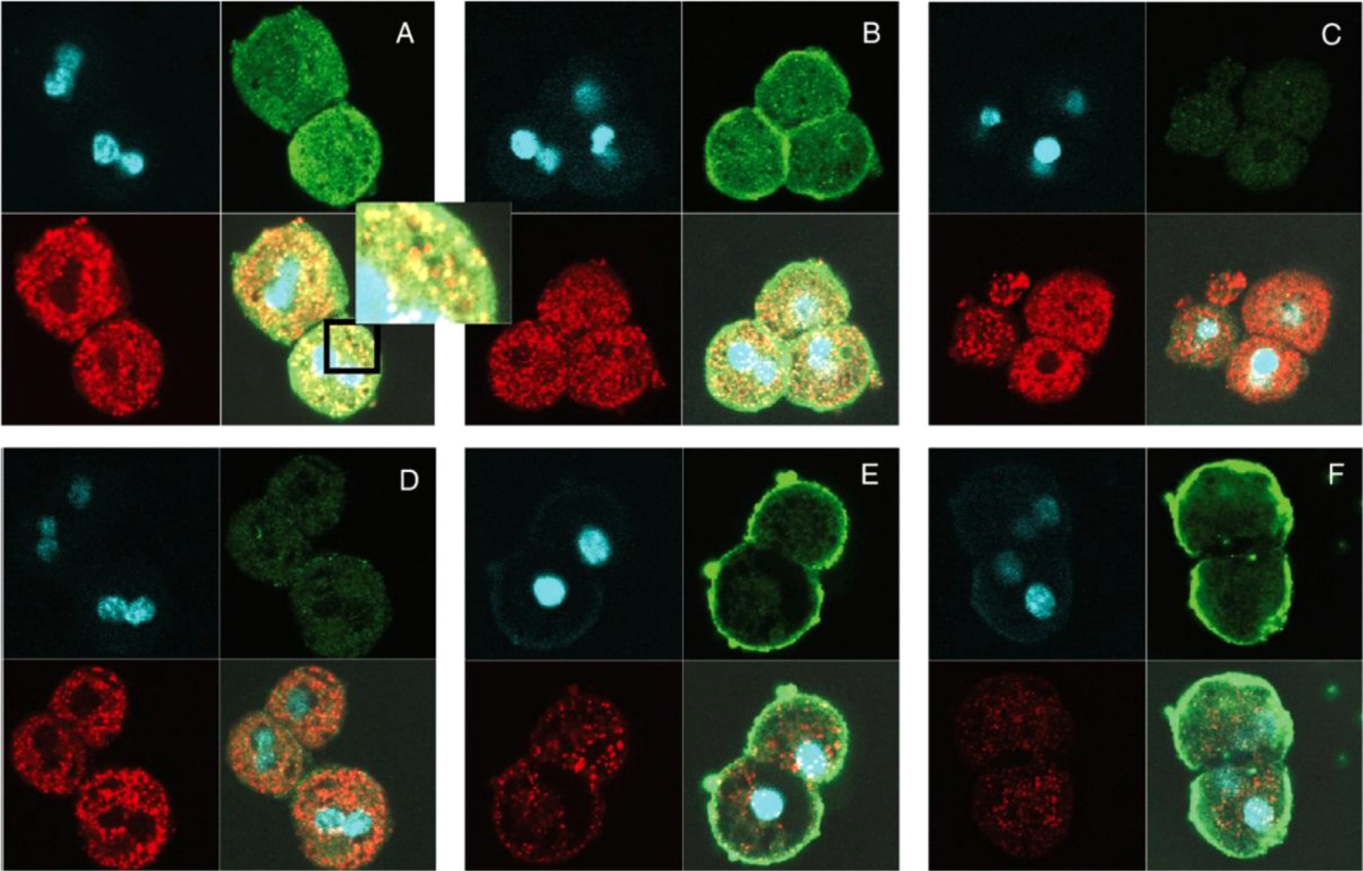
Representative confocal microscopy images of the colocalization of catalase (red stain) and protein-DMPO adducts (green stain) obtained by treating mouse hepatocytes (2.5×10^6^ cells/ml) with HOCl. (A) Cells were treated with three pulses of HOCl (20 μM, 30-min intervals) in the presence of DMPO; (C) same as A, but in the absence of DMPO; (D) same as A, but in the absence of HOCl; (E) same as A, but cells were obtained from catalase-knockout mice. Clockwise, the quadrants in each picture are laser microscopy showing DAPI (for nuclear stain), anti-DMPO stained with anti-rabbit (green/FITC conjugate); overlaid picture of laser microscopy obtained from anti-catalase and anti-DMPO (yellow shade obtained by overlaying red and green); laser microscopy showing anti-catalase stained with anti-mouse conjugated with rhodamine (12). (For interpretation of the references to color in this figure legend, the reader is referred to the web version of this article.)

**Fig. 4:**
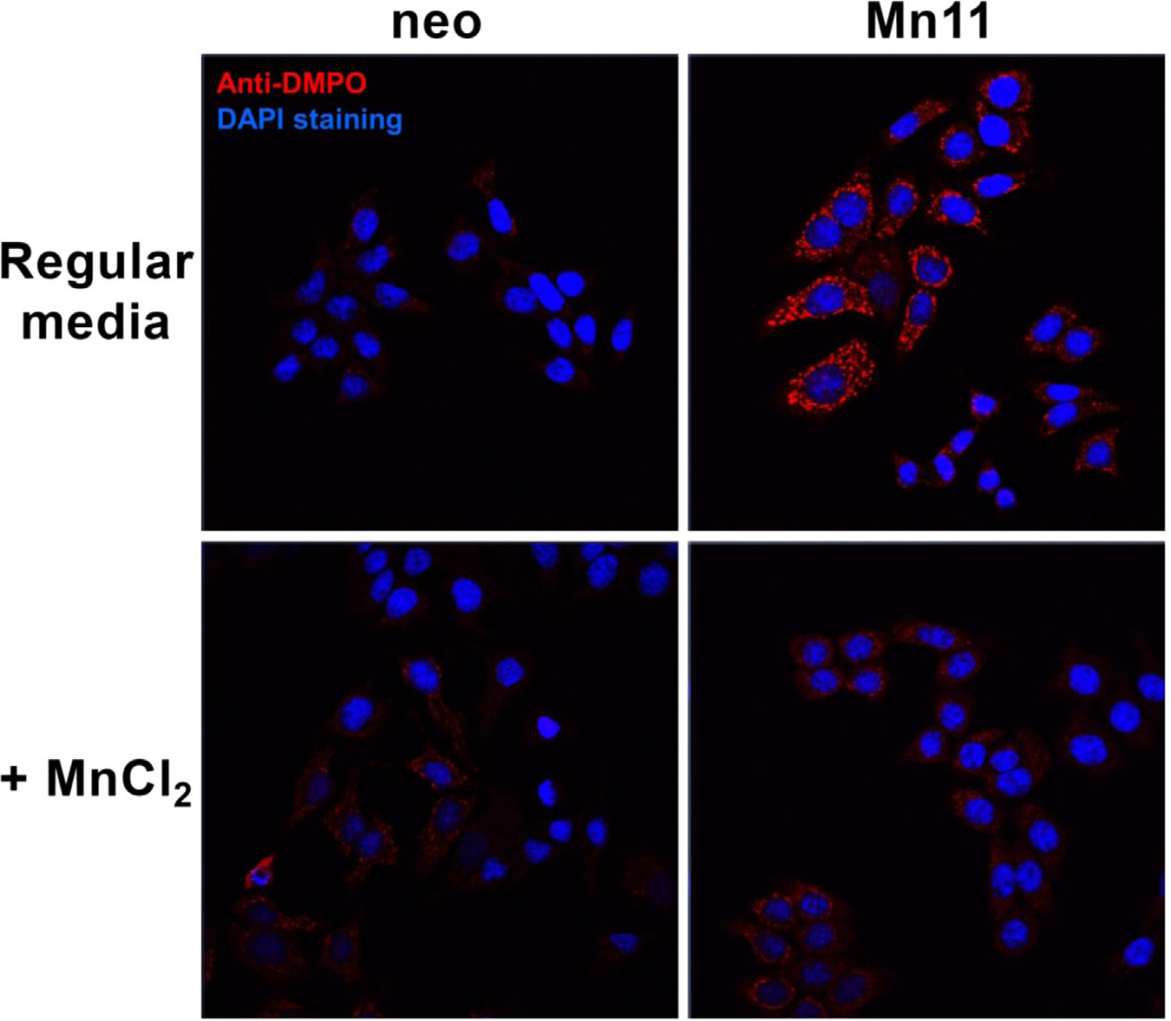
Cells were incubated with 40 mM DMPO, and then treated with paraquat (750 μM) for 10 h. Fluorescence immunohistochemistry was prepared for the detection of protein-centered radicals (anti-DMPO in red). Slides were covered with mounting media with DAPI (nuclear counterstaining in blue) just before confocal microscope imaging (13). (For interpretation of the references to color in this figure legend, the reader is referred to the web version of this article.)

**Fig. 5:**
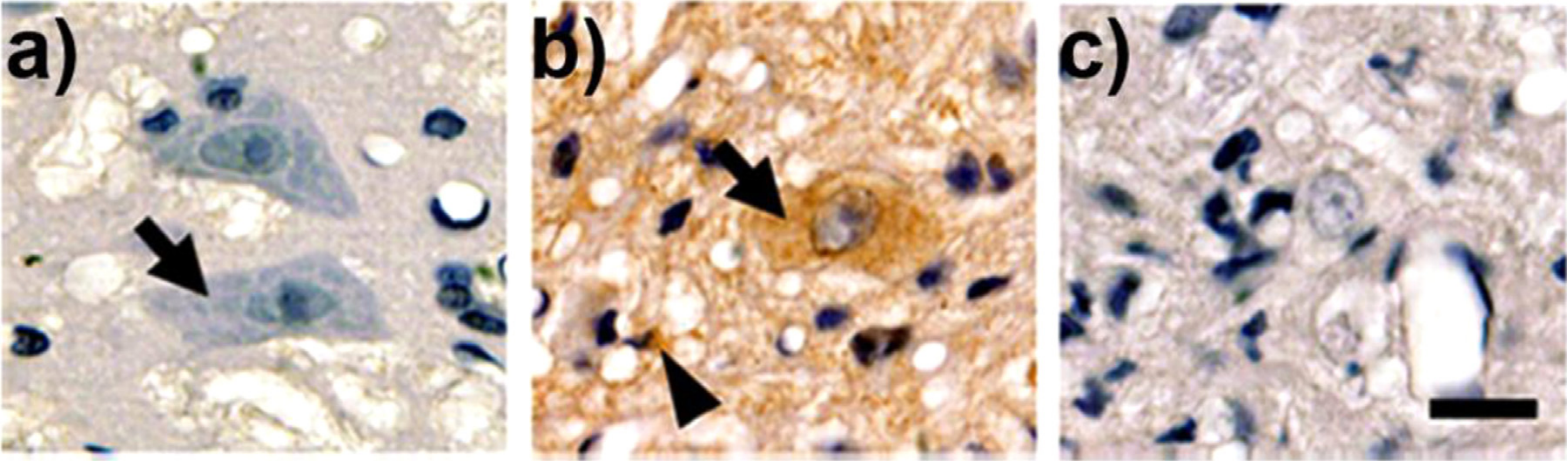
Immunohistochemistry in sections of the anterior horn of ***a***) DMPO-injected non-transgenic rats, ***b***) DMPO-injected early symptomatic SOD1^G93A^ rats, and ***c***) vehicle-injected SOD1^G93A^ rats. Note the intense punctuate immunolabeling in both motor neurons (arrows) and surrounding glial cells (arrowheads) in ***b*** compared with lack of labeling in ***a*** (14).

**Fig. 6:**
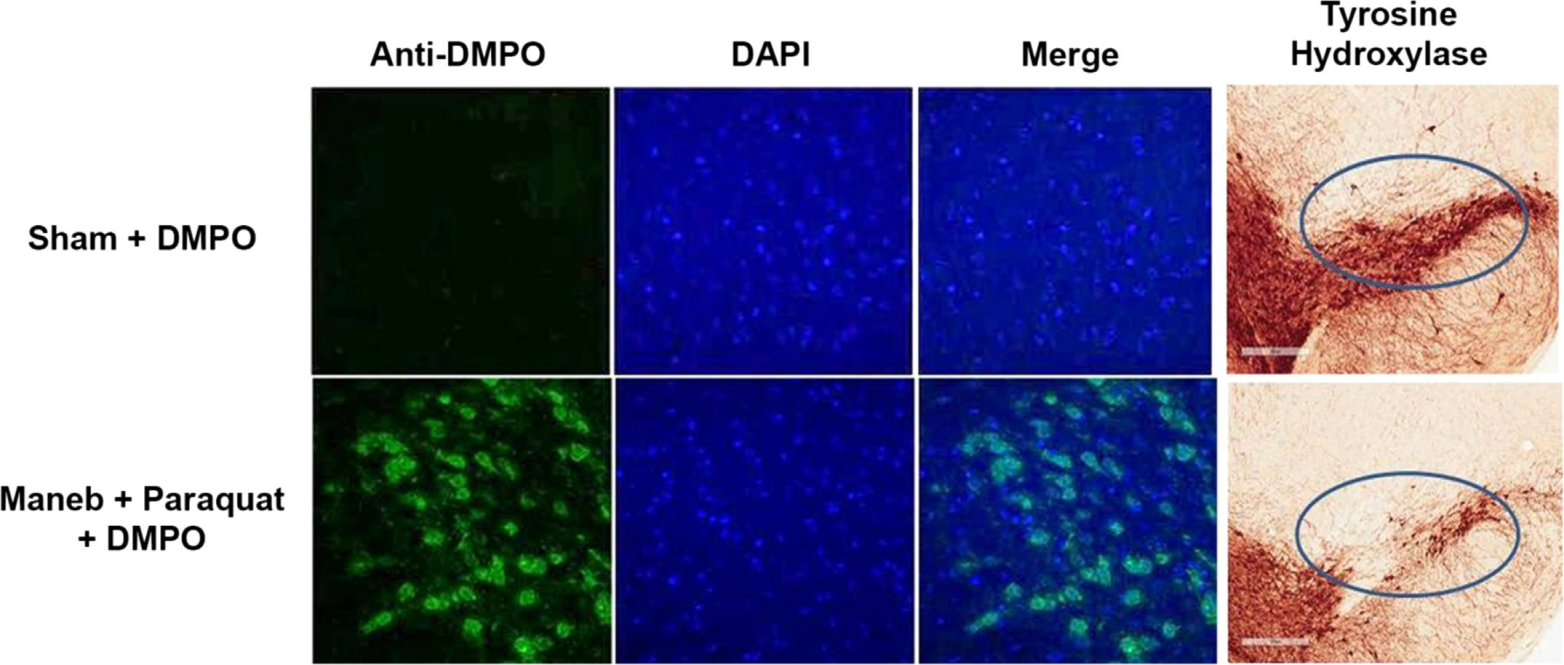
Confocal images of protein radical formation in the midbrain of mice after 6 weeks of Maneb (30 mg/kg, i.p.) and paraquat (10 mg/kg, i.p.) coexposure correlating with loss of tyrosine hydroxylase (7).

**Fig. 7:**
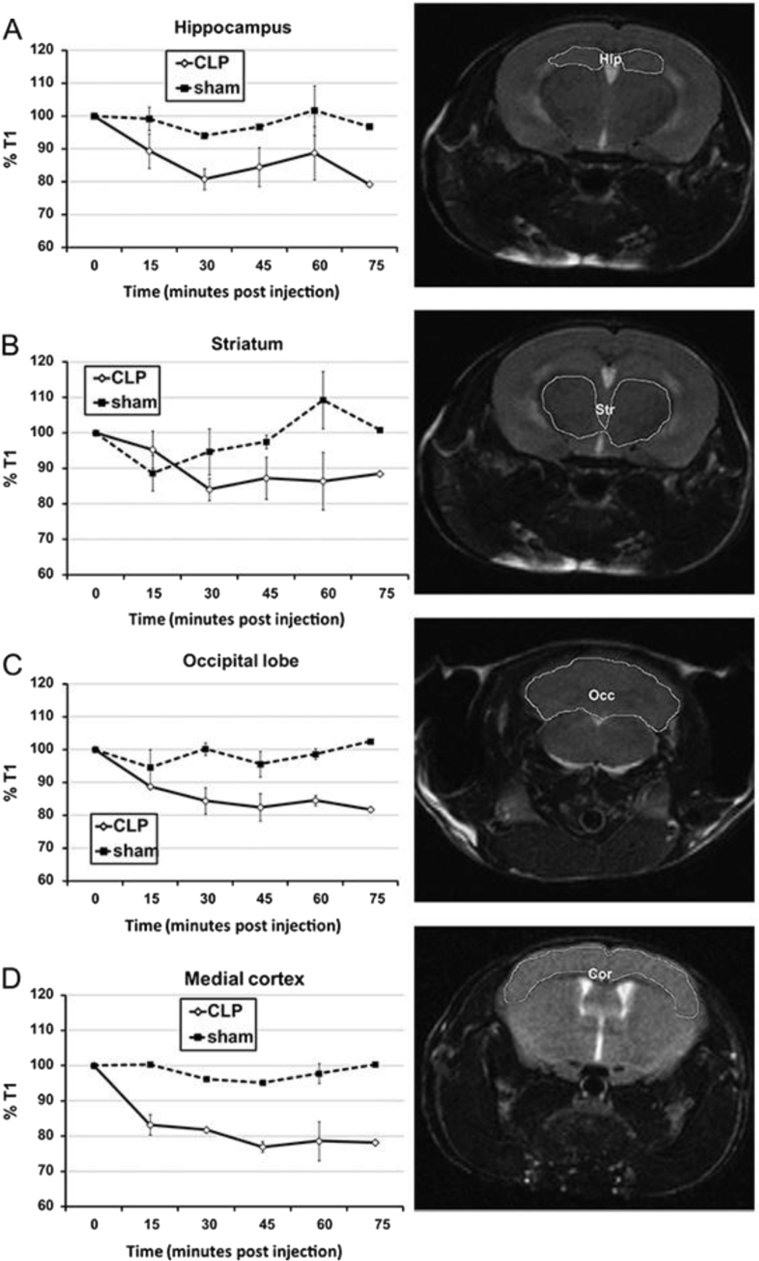
Kinetic distribution of the anti-DMPO probe in septic and sham mouse brain regions after cecal ligation and puncture (CLP). %T1 values at various time points are shown on the left, and outlined brain regions are shown on the right [normal mouse used for anatomical descriptions; (A) hippocampus, (B) striatum, (C) occipital lobe, and (D) medial cortex] (18).

**Fig. 8:**
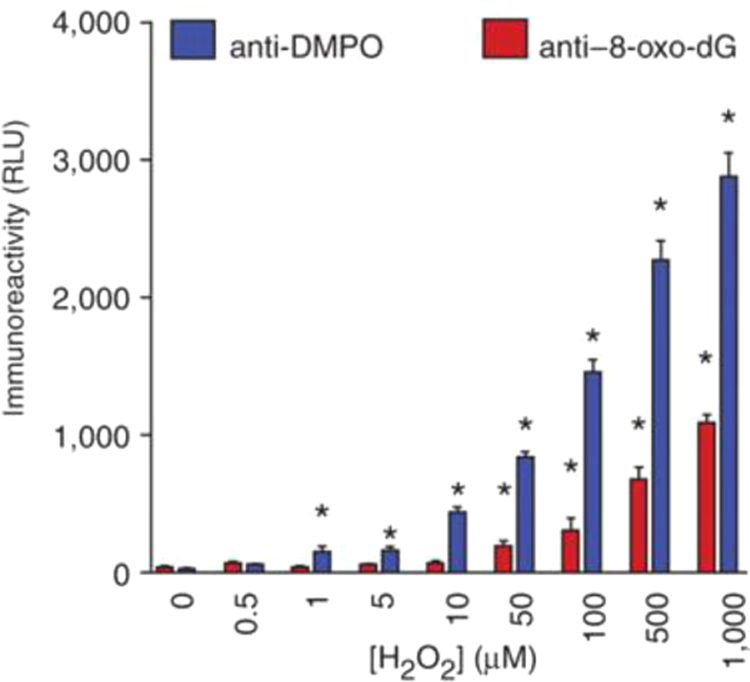
Detection of H_2_O_2_-induced, copper-catalyzed calf-thymus DNA nitrone adducts by immune spin trapping and 8-oxo-dG by ELIS (19).

**Fig. 9:**
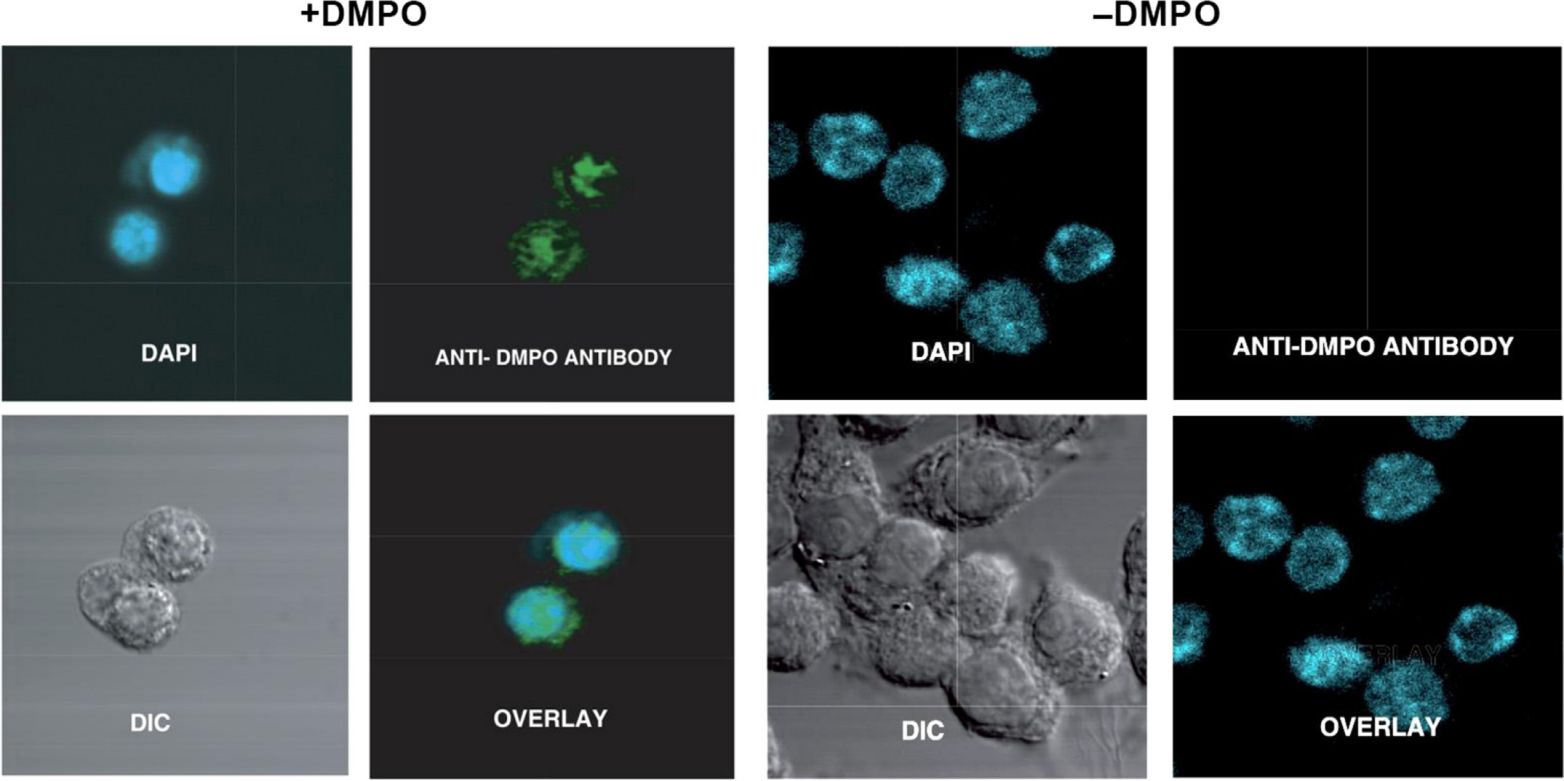
Confocal imaging of the free radical DNA in RAW 264.7 cells induced by Cu(II)/H_2_O_2._ Anti-DMPO immunoreactivity (green stain) could be seen primarily in the nucleus (DAPI), as evidenced by the colocalization. There was no DMPO immunoreactivity in cells that were not treated with the spin trap (20). (For interpretation of the references to color in this figure legend, the reader is referred to the web version of this article.)

**Table 1:** Advantages of free radical detection with electron spin resonance (ESR) – The Gold Standard.

• Direct ESR is physics – **No assumptions**! • ESR not only **detects** free radicals unambiguously, but can **identify** them • ESR is as sensitive as optical spectroscopy, but free radicals are very reactive **and insufficient concentration** is a major limitation • This **instability** of free radicals is **partially solved** by the organic chemistry trick of spin trapping • ESR is without question the **best way** to detect free radicals of small organic or inorganic molecules, such as **drugs or antioxidants**

**Table 2:** Limitations of free radical detection with electron spin resonance (ESR) – The Gold Standard.

• **Not sensitive** enough, especially for cell studies • **No subcellular location** of free radicals is possible • **No tissue distribution** of free radicals is possible except nitroxides at mM in vivo concentrations • **Very limited** number of free radicals detected in vivo • No **DNA-derived radicals**, except with **radiation and chemical** generation • ESR is relatively **expensive** • **Quantum mechanical** – higher math and physics
